# Perspective on Interdisciplinary Approaches on Chemotaxis

**DOI:** 10.1002/anie.202504790

**Published:** 2025-10-28

**Authors:** Juliane Simmchen, Daniel Gordon, John MacKenzie, Ignacio Pagonabarraga, Christina C. Roggatz, Robert G. Endres, Zuyao Xiao, Benjamin M. Friedrich, Tian Qiu, Kevin J. Painter, Ramin Golestanian, Claudia Contini, Mehmet Can Ucar, Gilad Yossifon, Jens Uwe Sommer, Wouter‐Jan Rappel, Kirsty Y. Wan, Judith Armitage, Robert Insall

**Affiliations:** ^1^ Faculty of Science University of Strathclyde Glasgow G11XL UK; ^2^ Universitat de Barcelona Institute of Complex Systems Universitat de Barcelona Barcelona 08028 Spain; ^3^ Faculty of Biology and Chemistry UFT Center for Environmental Research and Sustainable Technology University of Bremen Bremen Germany; ^4^ Department of Life Sciences & Physics of Life Network of Excellence Imperial College London London SW7 2AZ UK; ^5^ Physical Chemistry Technische Universität Dresden 01069 Dresden Germany; ^6^ German Cancer Research Center (DKFZ) Site Dresden 01307 Dresden Germany; ^7^ DIST (Interuniversity Department of Regional and Urban Studies and Planning) Politecnico di Torino Italy Milano Italy; ^8^ Max Planck Institute for Dynamics and Self‐Organization (MPI‐DS) 37077 Göttingen Germany; ^9^ Department of Life Sciences Imperial College London London SW7 2AZ UK; ^10^ School of Mathematical and Physical Sciences University of Sheffield Sheffield UK; ^11^ School of Mechanical Engineering Tel‐Aviv University Tel‐Aviv Israel; ^12^ Division Theory of Polymers Leibniz Insitute of Polymer Research 01069 Dresden Germany; ^13^ Department of Physics University of California San Diego La Jolla CA 92093 USA; ^14^ Living Systems Institute & Department of Mathematics and Statistics University of Exeter Exeter EX4 4QD UK; ^15^ Department of Biochemistry University of Oxford South Parks Road Oxford OX1 3QU UK; ^16^ Department of Cell & Developmental Biology University College London London WC1E 6BT UK

**Keywords:** Active colloids, Bacteria, Chemotaxis, *Dictyostelium*

## Abstract

Most living things on Earth – from bacteria to humans – must migrate in some way to find favourable conditions. Therefore, they nearly all use chemotaxis, in which their movement is steered by a gradient of chemicals. Chemotaxis is fundamental to many processes that control our well‐being, including inflammation, neuronal patterning, wound healing, tumour spread in cancer, even embryogenesis. Understanding it is a key goal for biologists. Despite the fact that many basic principles appear to have been conserved throughout evolution, most research has focused on understanding the molecular mechanisms that control signal processing and locomotion. Cell signaling – cells responding to time‐varying external signals – underlies almost all biological processes at the cellular scale. Chemotaxis of single cells provides particularly amenable model systems for quantitative cell signaling studies, even in the presence of noise and fluctuations, because the output, the cell's motility response, is directly observable. However, the different scientific disciplines involved in chemotaxis research rarely overlap, so biologists, physicists and mathematicians interact far too infrequently, methodologies and models differ and commonalities are often overlooked, such as the possible influence of physical or environmental conditions, which has been largely neglected.

## Graphical abstract

Chemotaxis is the movement of individual organisms towards or away from a chemical stimulus. This phenomenon is investigated in various disciplines, ranging from biology to physics and even engineering. This perspective aims to summarise recent developments in the field and encourage the unification of terminology to promote interdisciplinary knowledge transfer and collaboration.

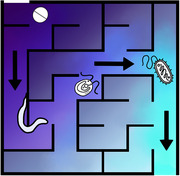

## 1 Historical Beginnings

The study of microbial behaviour and chemotaxis was made possible by the early discoveries in microscopy and microbiology made by the Dutch entrepreneur and lensmaker A. van Leeuwenhoek (1632‐1723).^[^ ^1^ ^]^ About a century later, T.W. Engelmann discovered bacterial aero‐ and phototaxis in 1883 by observing aerobic bacteria on the chloroplasts of algae.^[^ ^2^ ^]^ A few years later, W. Pfeffer observed bacteria and algae moving up nutrient gradients.^[^ ^3^ ^]^ Similar behaviours were subsequently discovered in other microorganisms, but technological innovations were lacking until the 1950s, when the link between chemotaxis and metabolism was demonstrated.^[^ ^4^ ^]^ Further developments in molecular genetics, the ability to engineer mutants and the resolution to visualise flagella made possible the crucial discoveries of Howard Berg and Julius Adler: the elucidation of the molecular mechanisms of bacterial chemotaxis.^[^ ^5^, ^6^ ^]^ For eukaryotes, after initial observations by Pfeffer, simple organisms such as protozoa and the amoeba *Dictyostelium discoideum* dominated early studies,^[^ ^7^ ^]^ which were then extended to other organisms, including neutrophils.^[^ ^8^, ^9^ ^]^ These classical studies of chemotaxis are complemented by, but rarely linked to, early observations and subsequent investigation of attracting or repelling chemical odours affecting complex eukaryotic organisms in the field of chemical ecology. Chemotactic behaviour was established to play a crucial role, for example, during foraging or mating of small organisms such as the domestic silk moth ^[^ ^10^ ^]^ and snails.^[^ ^11^ ^]^ Complex models have been developed mainly for prokaryotes, while studies of eukaryotic cells are often less mathematical.

In the early 2000s artificial agents showing responses to external stimuli (tactic particles) also entered the field. Evaluating the common grounds and differences among the three large categories (eukaryotic, bacterial, tactic particles), it becomes apparent that these agents must be different because bacteria and artificial active colloids move through liquid in a swimming motion, often exhibiting superdiffusive behaviour, while many eukaryotes move by adhesion‐based motility such as crawling.^[^ ^12^, ^13^ ^]^ Chemical‐induced movement has been documented for different cells though, including sperm and gametes of echinoderms,^[^ ^14^ ^]^ marine larvae of sessile organisms, and some larger multicellular invertebrates. Although the detailed mechanisms responsible for chemotaxis in different organisms are complex, the phenomenon emerges systematically. Using a theoretical approach, one can classify the phenomena into more systematic categories that describe the most general way that chemotaxis can occur. Due to the nature of decaying gradients (generally a rather slow decay), these interactions are often long‐range, and can be categorised into motions of five distinct groups. These include 1) speed depending on the chemical concentration (also often termed ‘chemokinesis'), 2) net drift along the gradient, 3) alignment‐ or polarity‐induced drift, 4) effective drift due to modulation of the rate of tumbles (as observed in bacteria like *E. coli*) and 5) anisotropic drift. These categories reflect the different underlying mechanisms that have been identified and the known responses to chemical gradients by microagents can be assigned to one of these.^[^ ^15^ ^]^ Linking the polarity response of the active agents to collective properties of the system bridges the gap between artificial and biological active matter. Different motility strategies may imply different gradient‐sensing strategies: An additional discriminating factor between prokaryotic and eukaryotic chemotaxis is that swimming bacteria must use temporal gradient‐sensing (because their body is too small to enable spatial sensing ^[^ ^16^ ^]^) while eukaryotic cells with crawling motility are thought to be able to use spatial comparison^[^ ^17^ ^]^ with a few notable exceptions.^[^ ^18^, ^19^ ^]^ It is also relevant to highlight the “level of interference” or “consciousness” to respond to a signal (or not). A fundamental difference is the fact that biological entities have evolved different sensing mechanisms, which include complex signal transduction pathways, while artificial systems (at least as of today) rely only on direct physical responses to stimuli.^[^ ^20^ ^]^ The input–output function (i.e. the stimulus–response relationship) is the culprit that biological cells can use to realise more complex functions than artificial systems, which may be overcome as soon as artificial systems become equipped with memory.

### 1.1 Recent Developments

Comparing the current state of the art with 20 years ago reveals some surprises, positive and unfortunate ones. Bacterial chemotaxis was, even then, more or less solved. Models are accurate and complete enough that quantitative disagreements with experiments generally reflect undiscovered biology rather than errors.^[^ ^21^ ^]^ The years have given understanding of more systems and better detail, but the theme has not changed. Eukaryotic chemotaxis is, on the other hand, no better understood than two decades ago; more pathways have been described, and more beautiful images captured by better microscopy, but there is still little fundamental understanding of how a cell can read a gradient or couple the resulting information to its movement. On a happier note, new topics have entered the discussion and changed its direction of the questions. It seems there is now a closer connection between living organisms and robotics or colloidal particles, which also opens the discussion to further points including ethics and the interface with artificial intelligence. Above all, however, it potentially allows chemotactic systems to be understood by synthesising them from scratch. New research concerning recent general information‐theoretic approaches (like infotaxis), and the many techniques that make up modern machine learning, shine light on a less biological angle, which goes hand in hand with the recent focus on fluid and mechanobiology in chemotaxis, and a growing emphasis on mechanical intelligence, linking the cell body to computation. And there is a growing body of work on communication networks – the model of a single cell chemotaxing in a steady state gradient is being superseded by a broader, more biological concept in which cells communicate with one another and with the environment, leading to complex and collective migration. Progress has been made to mechanistically understand the chemotaxis of sperm cells of marine invertebrates along helical paths, which represents an intriguing intermediate between temporal and spatial gradient‐sensing. This mechanism is more general and formally equivalent to phototaxis of swimming microalgae, yet still fundamentally different from the chemotaxis of eukaryotic cells with crawling cell motility.

An open question remains: which areas of the field have noticeably progressed from knowledge of facts to deeper understanding? This meeting suggested two things. First, that the underlying mechanisms by which cells chemotax have not been much better understood, though the profusion of new systems will surely help future research; and second, that our understanding of what chemotaxis can do, and where in biology it operates, is moving forwards rapidly.

## 2 Term Definitions

1. (Chemo)taxis: This taxis refers to the phenomenon in which the direction of an organism's motion is determined by the non‐uniform distribution of a physical quantity (i.e., the cell moves up or down a gradient of “chemical” quantity).^[^ ^22^ ^]^
2. (Chemo)kinesis: In contrast, kinesis occurs when an organism's speed, either translational or rotational, depends on the amount or intensity of a quantity, in this instance, chemical concentration.^[^ ^22^ ^]^

The definitions given are general, so they can also be adapted for further stimuli (e.g. photo‐taxis/‐kinesis). Some stimuli like gravity or electric fields are necessarily directional so there is only gravitaxis and galvanotaxis (but no gravikinesis).

### 2.1 Can These Definitions be Applied for Both, Artificial and Biological Systems?

It is commonly believed that taxis generally requires sensing, signal transduction, and a movement response.^[^ ^23^ ^]^ However, the question whether sensing is a crucial factor and how sensing is defined (in relation to information) is crucial. An example inspired by *Dictyostelium*: when a cell navigates & turns, the change in its direction or position alters its perception of signals; the statistics of this signal affect the response of the cell.^[^ ^24^ ^]^ Engineers call this information self‐structuring: the active motion defines the signal perceived.

Important questions include: Is “awareness”, or “consciousness” required in sensing? or can any reaction to chemicals already be defined as sensing? An important argument for this assumption is that molecular interactions are required for sensing and that would include thin layer interactions in a colloid. Which opens the pathway to the next question where to draw a boundary?

‘Is the number of states the difference? The number of states (or size of internal memory) might indeed be a key factor, as Nava et al.,^[^ ^25^ ^]^ Kromer et al.,^[^ ^25^, ^26^ ^]^ and many others have highlighted the importance of internal states, delays and hysteresis.

Many more questions can be asked, such as whether there is a grey area created by physical constraints (e.g. a motor that slows down under load)? Regarding the evolution and the origin of taxis: What is the simplest system capable of exhibiting taxis, and could taxis have already emerged in a prebiotic environment?

## 3 Areas that Will Benefit from Closer Collaboration

Many modern scientific questions are too complex to be solved by one discipline alone, and understanding chemotaxis is certainly one of them. When collaboration is motivated by the research question ‐ rather than the research area – different approaches, technologies and expertise need to be shared. One point to start with is to collect parameters and language and 1) unify or 2) translate them. We have identified several areas that would benefit from closer collaboration in order to improve the resolution of complex research questions:

- Experimental designs,
- Parameters and reporting styles,
- Models.

In the following paragraphs, we will summarise the key points we identified during the meeting and provide guidance for scientists entering the research area.

### 3.1 Experimental Setups

Experimental plans and descriptions of results vary strongly depending on the size and scale of the investigated actor, signalling and search strategies. However, sharing specialised tools and techniques across disciplines will mutually enhance our research. At the microscale where a continuous diffusion gradient builds up over time, microswimmers such as bacteria can rely on constantly increasing signals for their search strategies. At larger scales where organisms often find interrupted traces, search strategies adapt over the course of evolution to optimise results.^[^ ^27^ ^]^

Typically the main steps of experimental setup include preparation, assay assembly by transferring the active matter in question to specific locations and finally counting and/or tracking of the organisms followed by data analysis.

**Figure 1:**
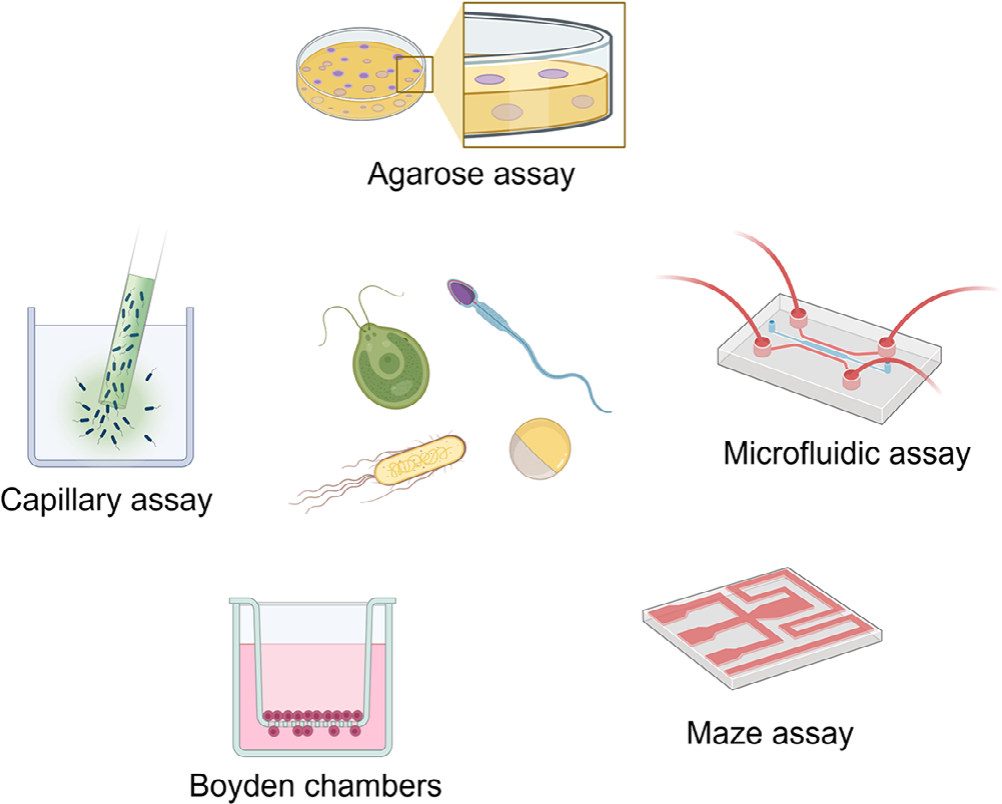
Different experimental setups enable the study of chemotaxis for different active agents. (Created in BioRender by Xiao, Z.)

For individual organisms, researchers have started attempts to unify the experimental approaches.^[^ ^28^, ^29^ ^]^ Obviously, these differ strongly between different organisms, as well as chemoattractants or chemorepellents, the nature of the medium (gel or liquid) as well as time scale and the required stability of the gradient.^[^ ^30^ ^]^ Some frequently used setups include agarose assays, gradients established by micropipette/hydrogel, transwell chambers, μ‐Slide assay, capillary assay, fluidic gradient (chips, T‐maze or maze including flow control^[^ ^31^ ^]^), and more specific methods such as printing 3D gradients by light using caged compounds.^[^ ^32^ ^]^ Also on the macroscale Y‐Maze/divided laminar flow cabinets are frequently used to observe chemotaxis for larger organisms (see also Figure 1).

Most studies employ setups with a “static” gradient condition experimentally defined by the concentration of the chemical stimuli source. The concentration used is often chosen not at the threshold level but at much higher concentrations to work reliably for chemotaxis characterisation. This provides basic insights that can be represented in models, but poorly represents realistic environments as it neglects the more complex interactive effects at threshold level as well as impacts of dynamic gradient conditions. Abiotic conditions of experimental setups can play a vital role but are rarely monitored continuously. While temperature is often reported, parameters such as oxygen levels or pH, which can be substantially altered at micro‐scale through respiration and metabolism processes even in a buffered system, are frequently neglected despite their potential to directly impact the organism as well as the chemical stimulus. Including these measurements on a regular basis would further benefit modelling and facilitate the step towards more complex models that take environmental conditions into account.

### 3.2 Parameters and Reporting Styles

Collaboration across different research fields improves parameterisation and reporting because explicit and precisely defined variables are required for mutual understanding. Parameters that are regularly measured and evaluated are **accuracy**, or the alignment of the motility with the direction of the gradient, **persistence**, or tendency to maintain the current direction, as well as **speed of cell motion**.^[^ ^29^, ^33^ ^]^ In order to create some more comparable values that characterise the chemotactic performance of a specific organism, specific parameters have been used.

**Figure 2:**
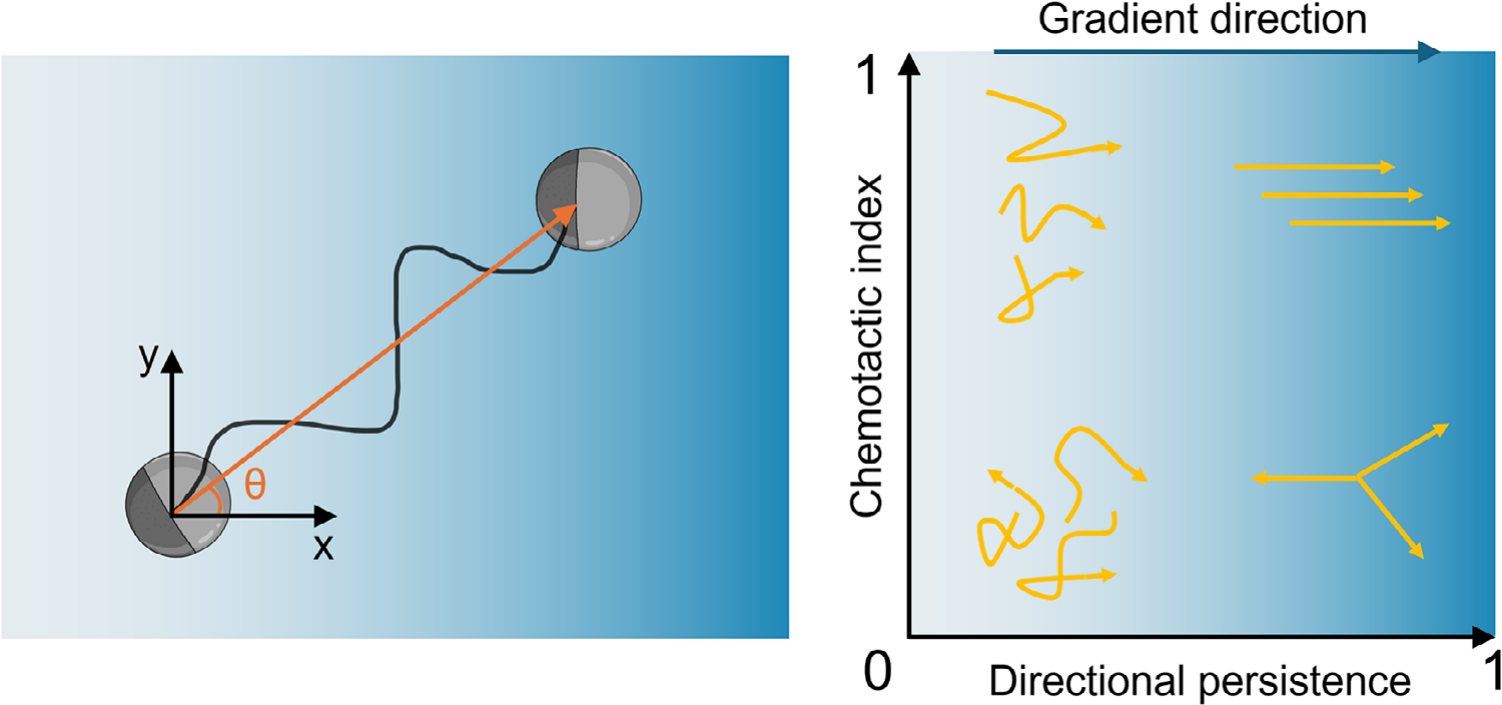
Left: Cell displacement forms an angle θ with the direction of the gradient. The CI is the ratio of displacement towards the gradient (displayed in orange) relative to the total path length (displayed in black). The directional persistence (DP) is defined here as the ratio of the total displacement to the total distance travelled. Right: High CI values are indicative of cell movement in the gradient direction, whereas high DP values are indicative of straight cell movement in any direction, Created in BioRender. Xiao, Z. Inspired by ref. [29].

- Chemotactic Index (CI), also sometimes referred to as chemotaxis index, calculated as the ratio of displacement towards the gradient (displayed in orange in Figure 2) relative to the total path length (black in Figure 2), with values ranging from +1 (indicative of positive chemotaxis) to –1 (representative of negative or anti‐chemotaxis^[^ ^29^, ^34^ ^]^
- Directional Persistence (DP) is the ratio of total displacement to the total distance travelled.
- Persistence length (*L*_*p*_)‐ Persistence length reports the characteristic distance a cell travels before randomising its direction.
- Drift velocities are occasionally reported for swimming bacteria and refer to a net velocity component of bacterial movement in the direction of the chemical gradient.

A more uniform reporting style, including comparable parameters and names, more cohesive setups and parameter reports will facilitate the use of data and increase comparability.

While for artificial swimmers the orientation is normally reported, this is rarely the case for bio‐swimmers. Would it be beneficial for the investigation of any of the bio‐organisms to consider orientation? An additional question is whether there is a clear “orientation” for the bio‐organism in question, and how to define it. For bacteria the axis defined from the main body to the flagellum can be tracked, while for amoebae this might require much more computational power to detect and report orientation. Re‐connecting to the experimental setups above, the environmental conditions used throughout the assay (including their stability over time) are pivotal and their reporting needs to be improved and unified.

### 3.3 Models

Chemotaxis models span from mathematical to chemical and/or physical, providing alternative abstractions of reality. These include those at scales from the molecular and mechanical regulation of movement responses to the collective movement dynamics of large populations of cells or organisms. Of the latter, the Keller–Segel (KS) model remains a popular framework after more than half a century. ^[^ ^35^, ^36^ ^]^ Formulated as a system of coupled partial differential equations, for a chemotactic population and its chemattractant described by density and concentration distributions, it was originally motivated by the classical phenomena of social aggregation in *Dictyostelium discoideum* and travelling bands formed by *E. coli;* ^[^ ^5^ ^]^ subsequently, it has been extended and applied—often in conjunction with experiments—to problems that range from tissue development and repair, disease and ecology.^[^ ^37^ ^]^

The KS model offers a phenomenological description of chemotaxis at the macroscopic level of large populations. When derived, relatively little was known of the microscopic processes by which cells detect and respond to an external attractant: for example, whether a cell explicitly computes a spatial gradient, a temporal gradient, or just the absolute concentration. As experiments have steadily revealed this detail, a growing spectrum of individual‐ or agent‐based models (ABMs) have arisen, which facilitates the inclusion of specific microscopic rules and the fitting to experimentally‐derived data. Stochastic random walk models range from particles that “hop” site to site on a fixed lattice to those that move continuously through space while undergoing velocity changes,^[^ ^38^, ^39^ ^]^ and each particle can be equipped with a representation of its chemosensory response: for example, the processes from receptor binding of external attractant to flagella activity in bacteria. “Multiscaling methods” can then connect these models to an approximating continuous form – often of a KS‐like form – that draw lines from microscopic signalling to population behaviour.

ABMs form a much broader group than just random‐walk models, covering an enormous spectrum of complexities.^[^ ^40^, ^41^ ^]^ Pointwise descriptions, which include many random walk models, restrict each agent to a single point, e.g., Langevin equations for the equations of motion of a centre of mass. Active Brownian particles (ABP), a standard model in the statistical physics of active matter allow for analytical solutions in activity gradients. Here, the role of connectivity and active polymers for taxis have been demonstrated. In particular it can be shown that active polymers display spontaneous taxis in gradients of activity without the need for information processing and signal transduction.^[^ ^42^ ^]^ Especially the sensing mechanism, how the lack of signaling capabilities is compensated for by direct physical response to stimuli has been frequently debated in artificial active matter.^[^ ^43^, ^44^ ^]^ To the best of our knowledge, chemotactic behaviour in active matter has only been observed as response to fuel concentration, allowing for a direct link between autophoretic propulsion mechanisms and chemotaxis. Yet rather unexplored are the factors that determine whether the result of these mechanisms will yield negative or positive chemotaxis.^[^ ^45^ ^]^ Various open source toolkits are now available that allow ABM models to be developed for cell (and animal) populations,^[^ ^41^ ^]^ many of which can be easily be tailored to include chemotaxis type responses.

At the other end, models can be formulated that include a geometrically complex and deformable body shape complete with internal structure. The intricacy of these models necessitates that their study is constrained almost exclusively to computer simulations, as well as the fitting of a large number of parameters. Nevertheless, cheaper computational time has widened their access and the level of detail they provide can be irresistible. Detailed models of biochemical chemotactic signalling exist for swimming bacteria such as *E. coli*, but are still scarce for other cells. Two past successes, for example, show the ability of mathematical and computational models to explain biological phenomena in terms of underlying principles – Neilson's description of biased, splitting pseudopods in terms of Meinhardt equations, and Arrieumerlou's ability to explain neutrophil steering in terms of quantized, biased extrusion of actin portions.^[^ ^46^, ^47^ ^]^

Modelling, therefore, can provide a valuable complement to experimental studies into chemotaxis. Yet, the classic modelling challenges persist: What is the most appropriate modelling framework and appropriate level of detail to choose? How do we find the right compromise between real‐world complexity and model simplicity? How do we parameterise a model when experimental data is not available? Modellers need to accept that useful models can only arise through an active engagement with the type of controlled experiments that can provide a rigorous groundtruth for a model. At the same time, experimentalists need to accept that models should start simple and only grow complexity as required. The aim of modelling is to test specific hypotheses about mechanisms, not to provide a complete description of the entire complexity of biology. In short, active interdisciplinary dialogue and collaboration is essential.

## 4 Open Questions/Challenges

The research field is dominated by rather simple systems (because they are easier to handle and study) but they do not allow answering the big questions. It is important to establish, strengthen and maintain the connections between the experimental, mathematical and the theoretical/computational soft matter community to increase the chances of solving the following complex problems:

1. What is the smallest level which can be called chemotaxis? Enzymes? Or smaller particles on the molecular level? The ability of biomolecules to self‐propel has been the matter of debate for almost a decade now. Although flow conditions are similar, scaling down the active object introduces constraints. First, the influence of Brownian motion increases, meaning the probability of actual ballistic behaviour decreases. Also, optical microscopy cannot be used for detection and documentation, so other evaluation methods are required, such as laser scattering or Malvern NanoSight.^[^ ^48^ ^]^ This problem is exacerbated if the nanoscale objects do not strongly scatter light, as is the case with enzymes. Further method development is needed for speed quantification, as opinions on enzymes as moving entities diverge strongly. Theoretical work^[^ ^49^, ^50^ ^]^ backed up by experiments^[^ ^51^, ^52^ ^]^ found enhanced diffusion for a variety of enzymes but there are doubts about the validity of the employed methods.^[^ ^53^ ^]^ Additionally, enzyme‐decorated particles are currently the subject of intense interest as enzymes could “bridge the gap” between living and nonliving active matter systems^[^ ^54^ ^]^ with some systems on their way to becoming relevant for biomedical applications.^[^ ^55^ ^]^
2. What are general interactions in (complex) natural systems?
  – If an organism is surrounded by a rapidly changing environment, how will it react? In general, models require more robustness to different environments, which then depends on the availability of good data (cell tracks, morphology and force measurements).
  – How realistic are findings we observe in the lab in constructed cases, such as aerotaxis?
  – Optimality of chemotaxis, e.g., with respect to time‐varying concentration fields is discussed without access to physiologically relevant concentration fields – how can we measure these parameters?
  – Which role does the environment play in mediating chemotaxis? Which environmental impacts affect chemotaxis?
3. Sensing and Detection
  – While eukaryotes detect spatial gradients by comparing receptors at different locations along the cell, bacteria mostly detect temporal variations.^[^ ^56^ ^]^ Can we generalise the certain observations such as the comparison temporal vs spatial position evaluation?^[^ ^57^, ^58^ ^]^
  – Another interesting trade‐off: exploitation versus exploration of information gain^[^ ^59^ ^]^ and the question how universally this can/should be applied?
4. Question of intention and origin (path differences between objects and living things)
  – Does the study of colloidal chemotaxis simplify the phenotypic diversity which is inherent in all biological entities?
  – How does biological chemotaxis compare to synthetic systems – autophoretic mechanisms render the notions of “intention” slightly unnecessary?
  – For ciliates, the cilia are often both, sensor and actuators, so the question arises ‐ for the earliest cells which were very minimal, how did they start to evolve such organs?
5. How valid are some of the following fundamental assumptions?
  – For colloids, does the reorientation mechanism must arise as part of its self‐propulsion?
  – Does the study of colloidal chemotaxis simplify the phenotypic diversity which is inherent in all biological entities?
6. How can we unify concepts that currently share a single name but refer to different processes (and vice versa)?
7. How to achieve better integration of experiments and modelling/theory?
8. How do collective behaviours emerge from individual chemotactic responses?
  – How well can discoveries made in single‐cell systems be transferred to chemotaxis in more complex multi‐cellular organisms?
  – Are externally supplied directional cues such as pre‐patterned chemotactic gradients so different from self‐organised guidance signals generated and shaped from within the system?
  – Do these heterogeneities lead to more complex patterns that require expanding theoretical frameworks?
  – How would large‐scale advective flows influence patterning?
  – How do interactions and phase segregation influence or even enable taxis?
    Here, it is important that certain phenomena can be mistaken for taxis. One recent example given by J. Moran et al.^[^ ^22^ ^]^ demonstrates that an interplay of chemokinesis and randomisation via rotational Brownian fluctuations, can lead to accumulation of active particles in regions of lower fuel concentrations. While this behaviour resembles negative chemotaxis, it is caused by motility‐induced phase separation. This example highlights how important clear definitions are, especially when bridging different classes of active agents.
9. What can AI contribute to the field?
10. How does chemotaxis study contribute to the benefit of human being?
11. Can scientists be encouraged to rank and quantify the parameters presented in the supporting materials of journal articles, so more reproducible studies and more comparison between conclusions for different works are possible?

**Possible pathways to overcome or solve these challenges** will require coordinated, interdisciplinary efforts. Wherever possible, interdisciplinary training (including AI literacy) for involved (early career) scientists should become the norm. This would enable an informed choice of setup or assay, as well as more general approaches to interpret data. Broadening the modelling approaches that experimentalists are familiar with, will enable different views on how to plan experiments and understand certain outcomes.

### Conclusions

We would like to emphasise that every time experimentalists and theoreticians are brought together, a previously unknown field of knowledge or expertise expands. Chemotaxis is a very timely and relevant example of a research area that benefits greatly from this approach, because the different approaches pursued in the different subfields can cross‐pollinate. Fascinating ideas, such as the influence of evolution, are unlikely to be discovered by any one group, while the inclusion of different disciplines can reveal much more about nature's approach to solving problems. Learning from and being inspired by nature has always been the path to success, so perhaps in this case too, understanding chemotaxis will allow us to solve many of the problems in processes related to chemotaxis.

## Conflict of Interests

The authors declare no conflict of interest.
